# Polymeric Emissive Materials Based on Dynamic Covalent Bonds

**DOI:** 10.3390/molecules27196635

**Published:** 2022-10-06

**Authors:** Shuyuan Zheng, Guofeng Liu

**Affiliations:** Shanghai Key Laboratory of Chemical Assessment and Sustainability, Advanced Research Institute, School of Chemical Science and Engineering, Tongji University, Shanghai 200092, China

**Keywords:** dynamic covalent chemistry, dynamic covalent bond, polymeric emissive material, stimuli polymer

## Abstract

Dynamic covalent polymers, composed of dynamic covalent bonds (DCBs), have received increasing attention in the last decade due to their adaptive and reversible nature compared with common covalent linked polymers. Incorporating the DCBs into the polymeric material endows it with advanced performance including self-healing, shape memory property, and so forth. However, the emissive ability of such dynamic covalent polymeric materials has been rarely reviewed. Herein, this review has summarized DCBs-based emissive polymeric materials which are classified according to the different types of DCBs, including imine bond, acylhydrazone bond, boronic ester bond, dynamic C-C bond, as well as the reversible bonds based on Diels–Alder reaction and transesterification. The mechanism of chemical reactions and various stimuli-responsive behaviors of DCBs are introduced, followed by typical emissive polymers resulting from these DCBs. By taking advantage of the reversible nature of DCBs under chemical/physical stimuli, the constructed emissive polymeric materials show controllable and switchable emission. Finally, challenges and future trends in this field are briefly discussed in this review.

## 1. Introduction

Polymers, such as plastic, rubber, fiber, and paint coating, are essential materials in our daily life [1,2,3]. Classical polymer materials that are mainly derived from covalent bonds are chemically inert, non-degradable, and insensitive to the surroundings, thus resulting in a fixed molecular make-up for these polymers, even under invasive stimulus, and limiting their dynamic properties. In addition, such polymeric materials suffer from both a low recyclability rate and unrepairable damage, which lead to an accompanying huge pollution of the environment. To address these problems, incorporating dynamic interaction into the polymer system becomes a good candidate. Dynamic interactions, including supramolecular interactions and dynamic covalent bonds (DCBs), can reversibly break down and build up, and well respond to external stimuli. Recently, dynamic interactions have been introduced into the polymer and endow the polymer with various advanced properties, such as self-healing, shape memory, adaptability, and stimuli-responsiveness [4,5,6]. Generally, there is a compromise between mechanical performance and dynamic behavior when dynamic interactions are introduced into the polymeric materials. Supramolecular interaction, possessing a low bonding energy (1–5 kJ/mol) [7], exhibits rapid exchanging character, thus endowing the polymeric materials with reversible regulation under ambient conditions. However, the mechanical properties of such polymeric materials are sacrificed, impeding the polymeric materials from being used in the engineering materials field. Compared to the weak supramolecular interactions, DCBs are types of bonding interactions with higher bonding energy. Once the DCBs being integrated into polymeric materials, the mechanical performance of such materials can be obviously improved and the dynamic behaviors of such materials can be feasibly regulated. Overall, DCBs combine the stability of covalent bonds and the adaptability of non-covalent bonds, thus enabling polymeric materials to be active when exposed to certain environment conditions.

Dynamic covalent chemistry, first proposed by Rowan et al., is related to chemical reactions carried out reversibly under conditions of equilibrium control [8]. In theory, all the chemical reactions are reversible to some extent and the reversible degree of the chemical bonds can be quantified by the thermodynamic equilibrium constant, *K*^θ^ [9]. When the *K*^θ^ of the chemical reaction is in the range from 10^−7^–10^7^, the reaction can be considered an irreversible one. Otherwise, the chemical reaction can be seen as reversible. DCBs, as the vital part of dynamic covalent chemistry, are usually stable in ambient conditions while also becoming reversible under external stimulus [10,11]. For constructing DCBs, the rational regulation of bond energy and modest reversibility of bonding reaction are feasible methods. For example, by introducing a large steric hindrance group, the reaction activation energy can be reduced and it promotes the covalent bond to be effectively reversible [12]. As a special type of covalent bond, DCBs can serve as chemical/covalent linkage to connect some structure elements to synthesize polymeric materials. Such polymeric materials can be divided into two parts: (1) amorphous polymer or gel, and (2) highly crystalline covalent organic frameworks (COFs). The DCBs in the polymeric materials can be easily tuned by endowing the polymeric materials with unique properties.

Polymeric emissive materials (PEM) have aroused increasing attention owing to their potential applications in chemical sensing, information encryption, bioimaging, and organic light-emitting diodes [13,14,15,16]. Compared to the small organic molecules, the superior processability as well as mechanical properties of polymers make them more feasible to fabricate devices. Moreover, polymer-based emissive materials can display signal amplification in contrast with the small molecule system [17]. Therefore, PEMs show various superior luminescent performances which the molecular systems do not have. However, polymers are usually robust and their structures are hard to regulate, thus greatly hampering the regulation of their emission behaviors. It is helpful to solve this problem by introducing DCBs into the PEMs since the DCBs are reversible under specific condition and can further modulate the inner structure of PEMs, which makes the obtained PEMs more tunable. Although the research on DCBs-based PEMs is relatively recent, increasing attention has been devoted to this field and considerable progress has been made. To our best knowledge, there are no corresponding reviews about such fascinating PEMs. Hence, we summarized the progress in this field to allow readers to gain a deeper understanding of the DCBs-based PEMs. To construct the PEMs derived from DCBs, two major strategies can be demonstrated: (1) The luminescent components (aggregation-induced emission luminogens, carbon dots, and quantum dots, etc.) as the fundamental units are merged into the polymer chain via DCBs, and (2) the luminescent elements are physically doped into the dynamic covalent polymers. Because of the dynamic nature of DCBs, the luminescent units can be flexibly incorporated into the polymeric materials and therefore show unique external stimuli responsiveness, making the polymers system display multiple emissive behaviors, including tunable emission wavelength, luminescence switch on/off, emission enhancement, etc. 

**Scheme 1:**
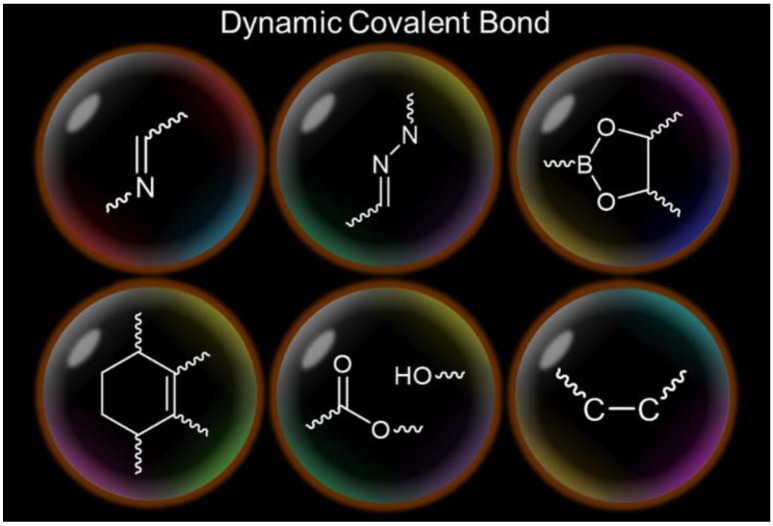
Dynamic covalent bond introduced in polymeric emissive materials.

In this context, we will highlight the PEMs consisting of various DCBs, and the PEMs will be divided into six parts which are classified according to the type of DCBs, including imine bond, acylhydrazone bond, boronic ester bond, Diels–Alder reaction, transesterification, and dynamic C-C bond (Scheme 1). These DCBs can be easily introduced into the polymer skeleton in either the main chains or the side ones. Due to their inherent dynamic nature, DCBs enable the polymer system with changing molecular dimension and result in a system with fascinating performance. For polymer systems, the performance resulting from DCBs might go far beyond the molecular dimension since polymers are composed of numerous repeat units which would greatly amplify the effect shown in one single molecular unit. Hence, in the beginning of each part, we will mainly demonstrate the chemical mechanism of the DCBs, following which the PEMs based on these DCBs and their performance are set out accordingly. Luminescent COFs, as a special PEM, have been widely reported, and there are several excellent reviews about the luminescent COFs [18,19]. Therefore, we will only selectively illustrate some representative luminescent COFs based on DCBs such as imine bond, acylhydrazone bond, and boronic ester.

## 2. Polymeric Emissive Materials Based on Imine Bond

**Scheme 2:**
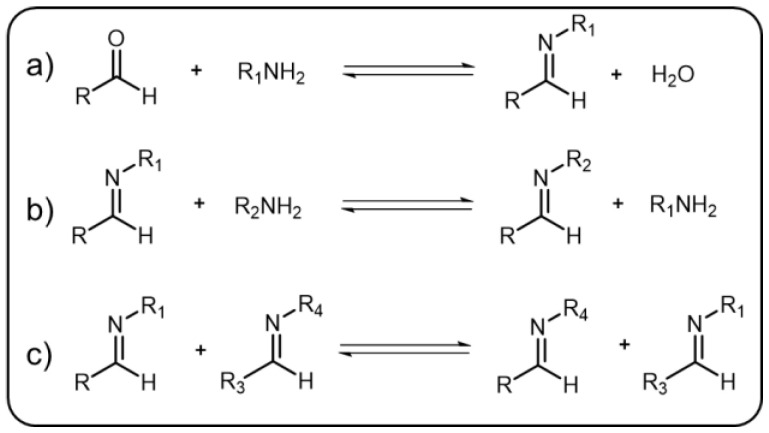
The three types of imine reactions: (**a**) imine condensation, (**b**) exchange, and (**c**) metathesis.

The condensation reaction, which takes place between amines and aldehydes to form imine along with a by-product of water, is a well-known organic reaction [20]. This reaction was named after Hugo Schiff, a well-known German chemist, as the Schiff-base reaction [21]. Generally, the by-product water formed in this reaction should be continuously removed since the enrichment of water would result in the equilibrium of the reaction being preferred to the opposite direction and facilitate the hydrolysis of imine. The pH value, similar to water, plays a key role for controlling the equilibrium of the reaction. In other words, intense acid pH can significantly affect the formation and break of the imine bonds, which are relatively stable under alkaline or neutral condition but hydrolyze under acid environment. Additionally, there are many other factors that can impel the reaction to go forward or backward. The equilibrium-controlled imine reactions have been divided into three types (Scheme 2) [20,22]: (1) Hydrolysis, in which the imine hydrolyze back to the precursor materials; (2) Exchange, in which another amine is introduced resulting in the two amines exchanging; and (3) Metathesis, in which upon introduction of a second imine, the two imines can undergo an exchange reaction of the amine to give a new imine. As a result, imine bond is considered as a representative DCB that can break and reform efficiently in a reversible manner. Due to the efficient reversible transformation, imine bond has been used as an ideal linkage as well as a stimuli-responsive element in dynamic polymers [23,24]. Other than being applied to polymers, imine-based systems are vital in supramolecular chemistry and materials science [25,26].

The reversible condensation of imine bonds has been widely used to fabricate numerous intrinsic self-healing polymers. Recently, some luminescent self-healing polymers based on the imine bonds have been reported. The Kuo group constructed several emissive composite supramolecular self-healing polymers [27], in which aminated polydimethyl siloxane (PDMS) combined with triformaldedhyde benzene and the methylene diphenyl diisocyanate to form a dynamic polymer network showing highly stretchable and effective self-healing performance, and perovskite quantum dots endowed the polymer with excellent emissive behavior. Utilizing boiling water to treat composite film for 2 min, the photoluminescence of the composite polymer only decreased by 16% from its initial intensity. It showed high-quality emission even in bending and twisting state, and the emissive color could be rationally tuned by changing the compositions of the perovskite quantum dots. Thus, highly stretchable performance, emissive stability, as well as tunable fluorescence of the composite polymer, make it an alternative to fabricate wearable emission devices. The Jelinek group also reported a luminescent self-healing polymer gel based on the carbon dots [28]. They prepared multicolor gels by reacting carbon dots (G-C-dot, B-C-dot, CoAP-C-dot) derived from different aldehydes with branched polyethlenimine (PEI). These carbon dots in gels maintained sufficient distance to block the aggregation-induced quenching, ensuring the effective emission of gels. The photoluminescent quantum yields of the G-C-dot/PEI gel, B-C-dot/G-C-dot/PEI gel, and CoAP-C-dot/PEI gel were 4%, 2%, and 1.9%, respectively. The Commission Internationale de l’Eclairage (CIE) chromaticity coordinates of the three gels were (0.25, 0.30), (0.34, 0.43), and (0.44, 0.46), respectively, which corresponds to cyan, green, and yellow emission. Stacking the B-C-dots/G-C-dots/PEI gel and CoAP-C-dots/PEI gel together, a white emissive gel could be obtained due to the self-healing process undergone between these two gels. Utilizing the multicolor feature of quantum dots, these two self-healing polymer systems could be used in optical devices, strain sensing, etc. 

**Figure 1:**
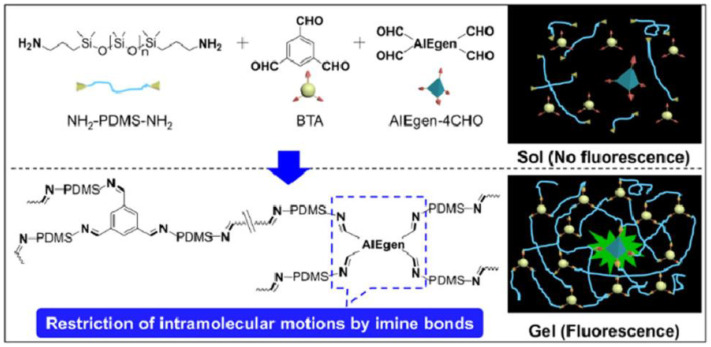
The formation of the emissive self-healing gel based on dynamic imine bonds and the luminescence mechanism in Ref. [29]. Reproduced with permission from Ref. [29]. Copyright 2019, American Chemical Society.

Apart from the highly luminescent quantum dots, the luminescent small organic molecules can also be used to fabricate luminescent self-healing polymers. Tang and coworkers reported a self-healing gel based on imine bond and the self-healing degree of such gel could be detected by emission intensity [29] (Figure 1). During the formation process of the gel, the dynamic imine bonds were formed continuously and the emission intensity of aggregation-induced emission luminogens (AIEgens) was increased gradually. The enhancement of the emission could be attributed to that the formed imine bonds restricted the motions of AIEgens. Taking advantage of such restriction induced emission, the self-healing process of such gels could be detected since the imine bonds were formed at the wound during the self-healing process. In this work, the dynamic imine bonds play a key role in the visualized microscopic processes for the self-healing gels. Following this work, Wang et al. utilized a similar method to construct a self-healing luminescent polymer gel, consisting of PDMS and a classic AIEgen (tetraphenylethene) [30]. Because of the efficient self-healing and emission of the two polymer gels, several blocks with different emission colors could be conglutinated together to fabricate the fluorescent codes with superior performance in anticounterfeiting. Utilizing imine bond as a reversible linkage, more and more polymeric materials with self-healing and emission features can be designed, and thus corresponding applications of such intriguing materials could be achieved.

In addition to the reversible condensation of imine bond, the exchange of imine bonds has been also used to regulate the emissive behavior of polymers. The Chen group reported that a polymer system could display a tunable fluorescence based on the imine exchange reaction [31]. Amine 4-(1H-phenanthro [9,10-d]imidazol-2-yl)benzenamine (L0) was chosen as the luminescent unit whose emission wavelength is located at 452 nm. Connecting 1,3,5-benzenetricarbaldehyde, aliphatic linear 2,2′-(ethylenedioxy) bis(ethylamine), and L0, a polymer film could be obtained which showed typically green emission. When the film was treated with aniline in MeOH, the imine exchange reaction undergone between the L0 and aniline and then the L0 released from the polymer film, enabling the emission color of the film gradually change from green to blue. These features of dynamic exchange emphasized the potential of dynamic chemistry for emissive materials.

The imine bonds can form under mild conditions and undergo reversible reactions under external stimuli. Owing to these properties, many dynamic materials have been fabricated via the imine DCBs. The Lehn group reported a dynamic self-sensing system by adding Zn^2+^ to the polyiminofluorenes [32]. In this work, a constitutional dynamic library (CDL) was selected, in which two amines and an aldehyde derivative were included. The polyiminofluorenes consisted of the amines and aldehyde. When Zn^2+^ was added into the polyiminofluorenes, the components of the polymer would exchange rapidly. With the gradually increasing addition of Zn^2+^, the polyiminofluorenes could quantitatively regenerate from one to another. The reason for the polymer constitutional regeneration within the CDL was that the preferential coordination of Zn^2+^ to the more basic aliphatic amine than to an aromatic amino group. These two polymers showed distinct emission wavelengths and intensity to display a self-sensing characteristic. Except for Zn^2+^, by tuning the pH of the imine-bond-containing polymeric system, the polymer constitution can be changed. Wang et al. reported a reversible pH-responsive AIE block copolymer via dynamic imine bonds [33]. The presence of imine bonds could make the AIE moiety be linked to the backbone of the copolymer. Due to the assembly nature of the copolymer in the organic solvent, regulating the pH of this system, the AIE moiety will reversibly associate and disassociate to the copolymer, leading to the reversible variation of the fluorescence intensity.

**Figure 2:**
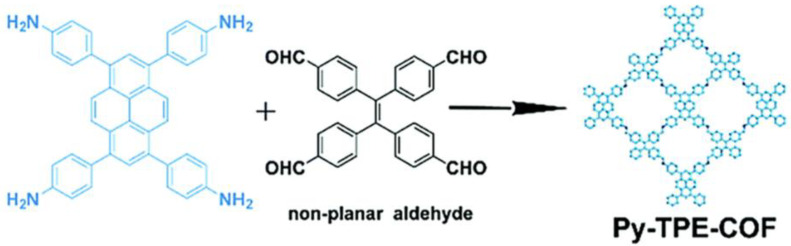
Imine-based 2D-COFs with high photoluminescence quantum yield in Ref. [38]. Reproduced with permission from Ref. [38]. Copyright 2018, Royal Society of Chemistry.

In addition to amorphous polymers, dynamic imine bonds have been reported to be the fundamental units to construct COFs [34], because the reversible dynamic nucleation and elongation processes are more feasible to stabilize the crystalline COFs. Some efforts have been devoted to fabricate imine luminescent COFs. Jiang and his coworkers reported four 2D-COFs, in which amino-modified pyrene and four different aldehydes were linked through imine bonds [35]. The Zhao group also reported several imine 2D-COFs which consisted of several aldehydes and the four amino modified tetraphenylethylene (TPE) [36,37]. Although the pyrene and TPE are excellent fluorophores, all the 2D-COFs showed no or weak emission, which could be attributed to the strong π-π interactions and the rotation of the imine linkages. After that, the Loh group reported a highly photoluminescent 2D imine-based COF (Figure 2) which consisted of non-planar TPE units and pyrene-based units [38] (Figure 2). The non-planar building blocks could reduce the π-π interactions of such COFs, and the COF could be processed into spherical nanoparticles which may restrict the bond rotation and reduce the π-π interactions of such a COF. These two factors should be the reason why the 2D COF shows high photoluminescence intensity. When dispersed in acetonitrile, the absolute fluorescence quantum yield of such COFs can reach 21.1%. 

## 3. Polymeric Emissive Materials Based on Acylhydrazone Bond

**Scheme 3:**
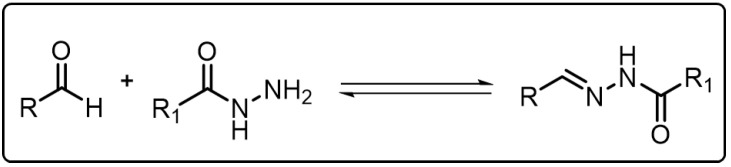
The formation of acylhydrazone via reversible condensation reaction.

Acylhydrazone bonds, which are structurally similar to the imine bonds, are generally formed from the condensation reaction of an acylhydrazine group and a carbonyl group as shown in Scheme 3 [20]. For acylhydrazone bonds, there are acylaminos adjacent to the C=N bonds, which decrease the electrophilicity of the C=N bonds, enabling the acylhydrazone bonds to be more stable than imine bonds even in the presence of water [39]. However, acylhydrazone bonds can also show reversible behavior as the imine bonds do. Under neutral and basic condition, acylhydrazone bonds are robust enough. When only catalytic amount of acid is added, the acylhydrazone bond can undergo a rapid bond exchange reaction similar to the imine exchange reaction. Besides acid, anilines and their derivatives can be also used as catalysts to accelerate the exchange or metathesis reaction of acylhydrazone bond even in neutral condition. By virtue of the adaptive nature of the acylhydrazone bond, it has been widely adopted in developing versatile dynamic covalent polymers [40].

**Figure 3:**
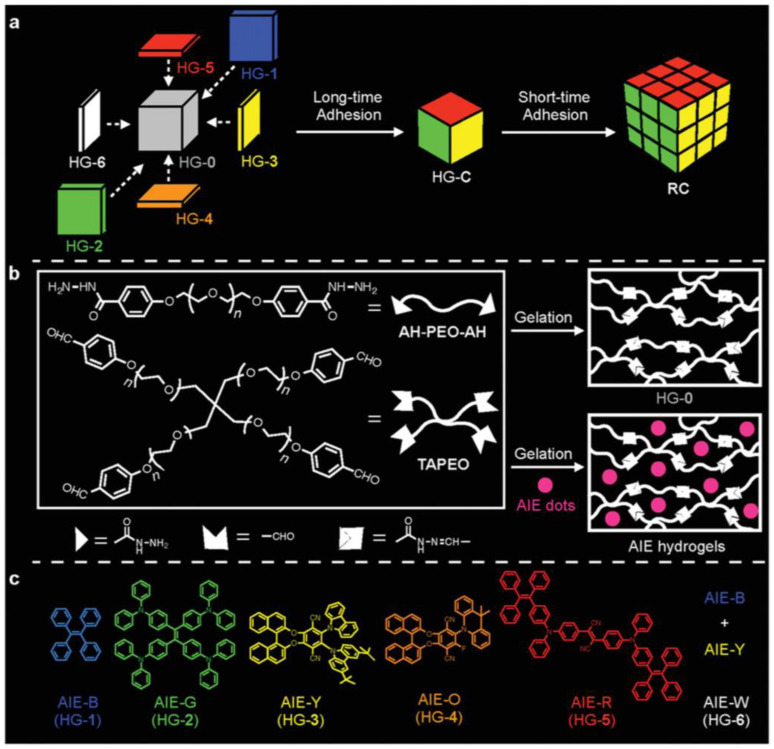
(**a**) Schematic diagram of chemical Rubik’s Cube (RC) preparation. (**b**) Chemical structures and representations of the hydrogels used in Ref. [44]. (**c**) Chemical structures of the AIEgens used in Ref. [44]. Reprinted with permission from Ref. [44]. Copyright 2019, Wiley-VCH.

The acylhydrazone bonds can undergo reversible bond exchange reaction, resulting in the structural diversity of the polymers linked by acylhydrazone bonds. The first example of acylhdrazone component exchange within polymers was reported by Skene and Lehn [41]. Following this work, more and more polymers have been built by using the reversible condensation reaction of acylhydrazone, in which the luminescent polymers were also included. In 2007, Lehn and his coworker reported the first luminescent polymer based on the exchange of acylhydrazone bond [42]. By attaching P1 and P2 together, the acylhydrazone bonds between these two polymers exchanged rapidly, accompanying the component recombination at the interface. The component recombination resulted in the extension of conjugation and further switched on the luminescence of the polymer whose fluorescent peak was located at the range of 450–600 nm. Later, they used the same reversible acylhydrazone exchange strategy for adjusting the fluorescence of glycodynamers [43]. Recently, the Tang group utilized the dynamic exchange of acylhydrazone for constructing a macroscopic luminescent “Rubik’s Cube” [44] (Figure 3). The Rubik’s Cube consisted of a hydrogel which was formed through reaction of a diacylhydrazine precursor with a tetraformyl counterpart. Building several block hydrogels and placing them in contact, they could be adhered together because of the acylhydrazone bonds exchange reaction undergone between these hydrogels. Over a short time, only a small percentage of acylhydrazone bonds undergone bond exchange reactions, which resulted in a low level of stickiness and the building blocks that adhered together could rotate under external mechanical force. By increasing the time, stronger interactions were expected, resulting in these building blocks forming robust hydrogels. Hence, AIEgen dots with different emissive colors could be capsuled into thin cuboid hydrogels, and then adhered to cubic hydrogels with long-term adhesion, to form cubic hydrogels showing multicolor emission behaviors. Adhering multicolor cubic hydrogels together for a short time, the “Rubik’s Cube” could be easily obtained. As mentioned above, by employing the efficient acylhydrazone bonds exchange reaction in the polymeric materials, the conjugated structure as well as the mechanical performance of the polymer can be flexibly regulated and be further used to construct PEMs. In that case, the authors deduced that it will be an important strategy to establish more interesting PEMs by tuning the dynamic exchange of acylhydrazone bonds in luminescent polymer. For instance, by introducing chiral units into such a system, tunable circularly polarized luminescence devices can be fabricated.

Additionally, the luminescent properties of polymers containing acylhydrazone bonds can be regulated under different chemical stimuli. The Hirsch group reported the pH-dependent morphology and optical property of a lysine-derived polymer [45]. Wu et al. developed a fluorescent polymer nanofilm based on acylhydrazone bonds [46]. The nanofilm could act as a fluorescent sensor with wide response range for real time detection of formic acid. When fumed by formic acid vapor, the fluorescence of the nanofilm was quenched. Due to the fast response (1.8 s) and high sensitivity of the sensor, it could be undoubtedly used to promote formic acid detection in various fields. Besides acid, metal ions can also be considered as the modulator to regulate the emission performance for polymers containing acylhydrazone bonds. For example, Fang et al. fabricated a tetraphenylethylene-based acylhydrazone gel for luminescence sensing of Cu^2+^ and subsequent CN^−^ detection [47]. Similarly, Wang et al. reported two crystalline polyacylhydrazone organogels whose fluorescence could respond to metal ions [48]. Very recently, the Tang group reported a phototriggered AIE (PTAIE) acylhydrazone gel which could be used for 4D soft patterns [49]. The PTAIE phenomena resulted from phototriggered coordination between the metal ion (Zn^2+^, Al^3+^, Ga^3+^, and Cd^2+^) and TPE-4SAH. TPE-4SAH was synthesized through condensation reaction of 4-methoxybenzhydrazide and TPE-cored-salicylaldehyde. Coordinating with different metal ions, the fluorescent colors of TPE-4SAH were varied. Taking advantage of the PTAIE, the TPE-4SAH was chosen as a model compound to construct an acylhydrazone gel. Once the metal ions were coated at the surface of the gel, under UV irradiation, the PTAIE occurred between the metal ions and gel. The radius of the metal ions could significantly affect the coordination process since different ion radius resulted in a different diffusion rate of the metal ions into the gel. Utilizing the different diffusion rates of various metal ions, time-dependent fluorescent patterns were realized through introducing different metal ions to the surface of the gel under continuous UV irradiation. These acylhydrazone-bonded polymers show various emissive characteristics under different chemical stimuli, in which fluorophores play a key role in the polymer systems. However, the polymers composed of non-emissive units but showing good emission properties are very rare. Very recently, Maiti et al. reported a luminescent polymer gel entirely consisting of two non-emissive units [50]. The emission of the gel resulted from the intermolecular H-bonding assisted aggregation.

**Figure 4:**
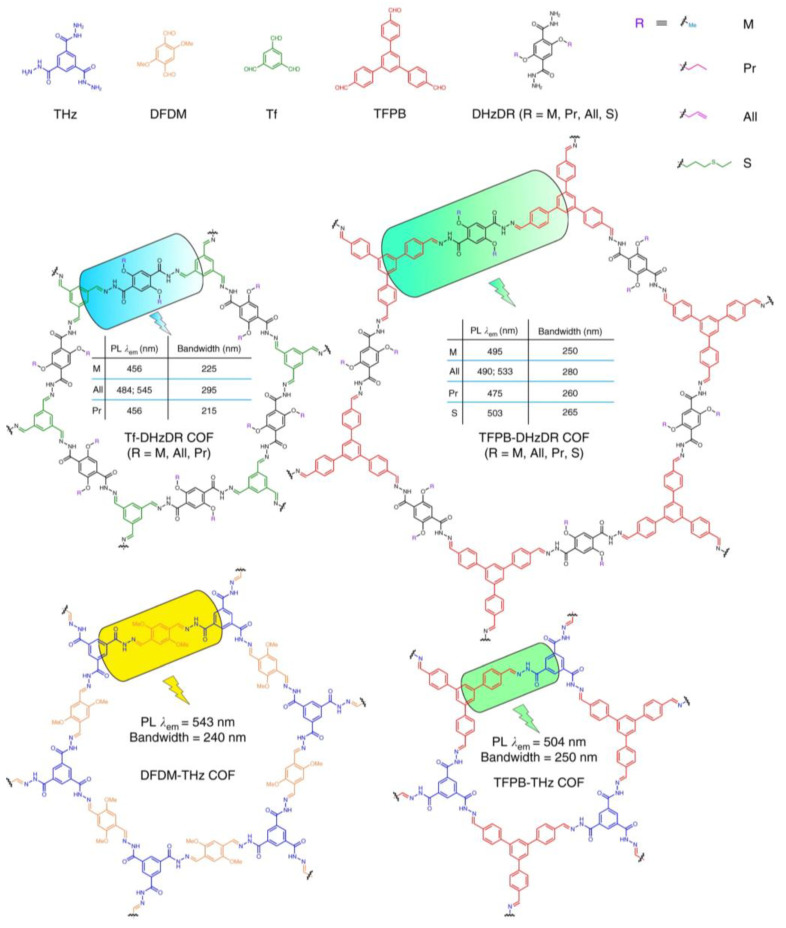
Synthetic scheme for accessing the emissive COFs based on acylhydrazone bond. The photoluminescent emission maxima and bandwidths are shown for each COF from Ref. [52]. Reprinted with permission from Ref. [52]. Copyright 2018, Nature Publishing Group.

Acylhydrazone bond is one of most important dynamic linkers to construct COFs. Compared to the rotatable imine bonds, the relatively rigid acylhydrazone bonds are more feasible to form luminescent COFs. In 2016, the Wang group utilized the 2,5-bis(3-(ethylthio)propoxy)terephthalo hydrazide and 1,3,5-triformylbenzene as the fundamental units to synthesize a luminescent COF, which showed blue emission both in solution and solid state [51]. After that, a series of acylhydrazone-based luminescent COFs were synthesized by Li et al. and they systematically studied the factors that could influence the emission performance of acylhydrazone-based COFs [52] (Figure 4). The intralayer and interlayer hydrogen bonds could restrict the rotation of intramolecular bonds and result in the photoluminescence of COFs. Finely tuning the corresponding components, the COFs showed multicolor emission from blue to yellow. However, the emission of the acylhydrazone-linked COFs could be partially quenched by photoinduced electron transfer (PET) effect. Hence, to enhance the emission intensity of the acylhydrazone-linked COFs, suppressing such transfer could be considered an ideal choice. For instance, Li et al. designed an acylhydrazone-linked TFPPy-DETHz-COF which showed weak green–yellow luminescence at 540 nm in tetrahydrofunan with an absolute fluorescence quantum yield of 4.5%. Adding the F^−^ into the suspension, the absolute fluorescence quantum yield of the COF increased to 17% and the lifetime increased from 1.4 ns to 2.7 ns, indicating that the presence of the F^−^ could effectively suppress the electron transfer process of TFPPy-DETHz-COF. Thus, the luminescence of the COF could be significantly enhanced [53].

## 4. Polymeric Emissive Materials Based on Boronic Ester Bond

**Scheme 4:**
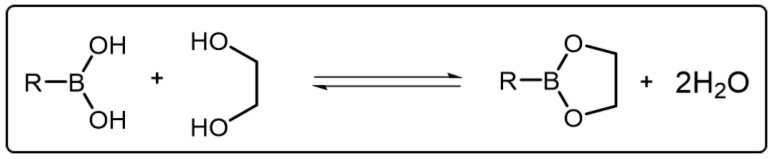
Reversible break and reform of the boronic esters between boronic acids and diols.

Boronic acid can bind with diol to give a cyclic boronic ester and additional water through a condensation reaction [54,55] (Scheme 4). In this condensation reaction, boronic acids act as Lewis acids to receive electrons while diols act as Lewis bases to donate electrons for producing boronic ester. The formation of boronic ester is usually in a reversible manner. For instance, in aqueous solution, when the pH value is above the pK_a_ of boronic acid, the equilibrium of reaction is biased to the formation of boronic ester. On the contrary, the equilibrium of reaction is biased to the hydrolysis of boronic ester [56]. Thus, the exchange of the boronic ester bond can occur associatively or dissociatively by tuning the pH of the reaction condition. In other words, the boronic ester bond is reversible in response to pH. Besides pH, the dynamic balance of boronic ester bond can be also regulated by other stimuli. Boronic ester bond, when embedded into polymeric materials, similar to other DCBs, can endow the materials with advanced properties owing to its adaptivity, which extends the application prospects of such polymeric materials.

Taking advantage of the pH sensitivity of boronic ester, Xu et al. reported an autonomous fluorescence regulation polymer system [57]. The autonomous regulation was controlled by a chemical oscillating reaction in which the pH of the system regularly changed from 3.68 to 9.06. The polymer was composed of poly(N,N-dimethyl acrylamide-b-4-(2-acrylamidoethoxy) carbonyl phenylboronic acid) (PDMA-b-PAPBA) and Alizarin Red S (ARS). ARS was emissive when grafted to the PDMA-b-PAPBA through boronic ester bond. Reducing the pH value, the ARS was released from the polymer, causing the fluorescent quench. Therefore, the polymer system displayed autonomous fluorescence regulation by chemical oscillating reaction since the dynamic boronic ester bond spontaneously breaks and reforms upon changing the pH.

**Figure 5:**
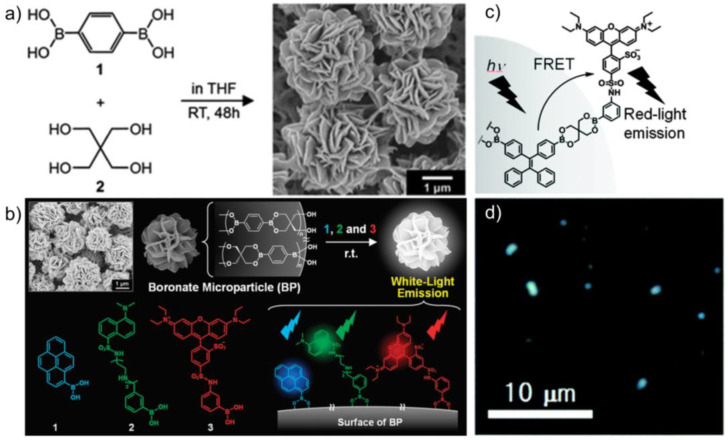
(**a**) The formation of flower like assembly in Ref. [59]. Reproduced with permission from Ref. [59]. Copyright 2012, Royal Society of Chemistry. (**b**) Preparation of white-light emitting boronate assembly. Reproduced with permission from Ref. [60]. Copyright 2013, Royal Society of Chemistry. (**c**) A plausible partial structure where fluorescence resonance energy transfer occurs in tetraphenylethylene and rhodamine B-based assembly. (**d**) The fluorescence microscopic image of white-light emissive assembly in Ref. [61]. Reproduced with permission from Ref. [61]. Copyright 2015, Royal Society of Chemistry.

Saccharides, containing multiple-hydroxyl groups, are often used to react with boronic acid to form the reversible boronic ester bond. Based on this, the Wang group reported a fluorescent probe for glucose based on the reversible boronic ester bond [58]. The bodies of the fluorescent probes were phenylboronic-acids-modified magnetic nanoparticles, and the fluorescent multi-hydroxyl carbon dots (fluorescence quantum yield up to 58.7%) were linked to the nanoparticles via the condensation reaction of the multi-hydroxyl groups and phenylboronic acids. Due to the light shielding effect of the black magnetic nanoparticles, the entire system appeared in non-emissive state. Adding the nanoparticle into the glucose solution, the fluorescence appeared since the multi-hydroxyl carbon dots which were emissive nature released from the nanoparticles. This system possesses a wide linear range (0.2–20.0 mM) and a low limit of detection (0.15 μM). Thus, it can be used for biosensors and environmental monitors. Except for saccharides, other molecules with multiple-hydroxyl groups are also ideal units to condense with boronic acid derivatives. Therefore, boronic-ester-bond-linked polymers which consist of different boronic acids and hydroxyl monomers can be full of variety. For example, the Kubo group developed a polymer microparticle (BP) through condensation of benzene-1,4-diboronic acid and pentaerythritol which could assemble into a flower-like morphology [59] (Figure 5a). They further grafted primary tricolor emissive dyes (1, 2 and 3) onto the BP [60] (Figure 5b). The CIE chromaticity coordinates of these three BP were (0.17, 0.04), (0.27, 0.44), and (0.61, 0.35), respectively. By co-grafting 1, 2, and 3 with BP and simply adjusting the relative concentration of 1, 2, and 3, white light emissive BP could be obtained and the CIE chromaticity coordinate of this BP was (0.31, 0.35). Utilizing the same strategy, Kubo et al. prepared a blue emissive boronate assembly consisting of di(boronic acid)appended tetraphenylethylene and pentaerythritol [61] (Figure 5c). Grafting rhodamine B onto the surface of the assembly, the assembly was endowed with white light emission owing to the fluorescence resonance energy transfer that occurred between the tetraphenylethylene and rhodamine B. Both the two white light emitting particles were formed through the bonding of fundamental units which contain the boronic acid and multiple hydroxyl groups, respectively. Hence, by expanding the category of the boronic ester monomers, such as multi-boronic modified chromophores, more luminescent polymeric materials can be developed. 

**Figure 6:**
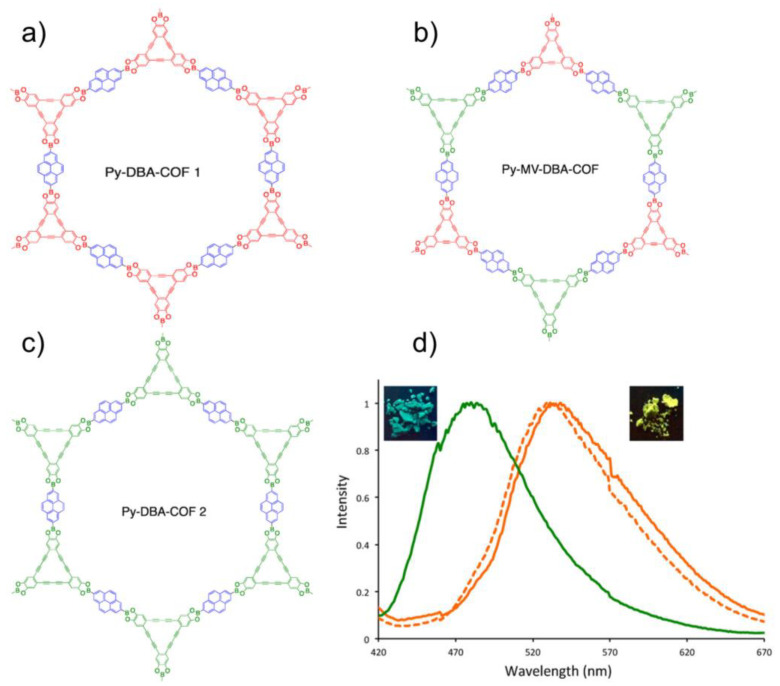
The chemical structure of (**a**) Py-DBA-COF 1, (**b**) Py-MV-DBA-COF, and (**c**) Py-DBA-COF 2. (**d**) Normalized emission spectra and photographs of Py-DBACOF 1 (orange line), Py-DBA-COF 2 (green line), and Py-MV-DBA-COF (orange dashed line), respectively. Reproduced with permission from Ref. [63]. Copyright 2016, American Chemical Society.

Boronic ester, similar to the imine or acylhydrazone bond, has been widely applied to construct COFs. The boronic esters serving as the COFs’ linkers are formed through the condensation of boronic acids and catechol derivatives. Introducing fluorescent units modified with such boronic acids and catechol derivatives, the luminescent COFs can be constructed. In 2008, Wan et al. reported the first example of a belt shaped luminescent COF, consisting of a typical fluorophore pyrene and triphenylene functionalities [62]. Utilizing the solvothermal method, the COF was synthesized through a condensation reaction of 2,3,6,7,10,11-hexahydroxytriphenylene and pyrene-2,7-diboronic acid. The COF could harvest a wide range of photons, due to the intramolecular singlet energy transferred from the triphenylene to the pyrene units, and showed bright luminescence. Since then, a series of luminescent COFs linked by boronic ester bonds have been reported. For example, the McGrier group constructed three luminescent COFs containing a homogeneous and heterogeneous distribution of dehydrobenzoannulene (DBA) vertex units [63] (Figure 6). These COFs were synthesized by condensation reaction of the pyrene-2,7-diboronic acid and different ratio of catechol modified DBA. The three COFs were named after Py-DBA-COF 1, Py-DBA-COF 2, and Py-MV-DBA-COF, respectively. Py-DBA-COF 1 and Py-DBA-COF 2 were formed via linking PDBA with DBA [12] and DBA [18], respectively, while Py-MV-DBA-COF was obtained by condensing PDBA with DBA [12] and DBA [18], in which the molar ratios of PDBA, DBA [12], and DBA [18] were 2:1:1. The Py-DBA-COF 2 showed a blue-greenish luminescent color and Py-DBA-COF 1 and Py-MV-DBA-COF were yellow emissive. Compared to the DBA [18]-based COFs, the lowest-energy transitions of the DBA [12]-based COFs were symmetry-forbidden in the ground state, which could be the reason why the emissive colors of these COFs were different. Besides polycyclic aromatic hydrocarbons like pyrene, AIEgens are also charming fluorophores with which to construct boronic-ester-bond-linked luminescent COFs. The Jiang group reported the first AIE-based COF which was formed by condensation of TPE-cored boronic acid and 1,2,4,5-tetrahydroxybenzene [64]. Incorporating TPE units into such COFs which possessed rigid environment could reduce the excitation-energy dissipation of the TPE units, resulting in high luminescence of the COF with the absolute fluorescence quantum yield up to 32%. In this regard, boronic ester bond can be considered a feasible linkage to binding more emissive elements for fabricating a series of luminescent COFs.

## 5. Polymeric Emissive Materials Based on Transesterification

**Scheme 5:**
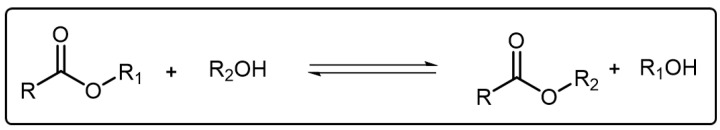
The representative reaction formula of transesterification.

**Figure 7:**
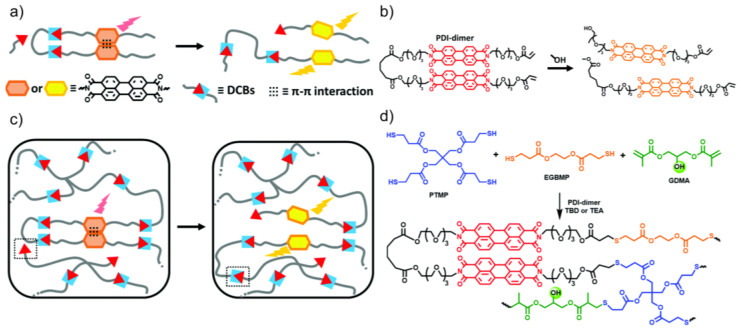
Schematic image of the color change mechanism of the folding fluorescent probe in (**a**) molecule and (**c**) polymer state. (**b**,**d**) are the molecular structures of the folding fluorescent probe in molecular and polymer state. Reproduced with permission from Ref. [69]. Copyright 2021, Royal Society of Chemistry.

Transesterification is a typical reversible reaction where an ester can transform into another through an associative and dissociative process of the alkoxy moiety and it has been widely used in laboratories and industries [65] (Scheme 5). In order to facilitate the transesterification reaction, elevated temperature as well as a catalyst are both necessary factors. In 2011, Leibler and coworkers developed a new kind of polymeric material which was later named vitrimer [66]. The vitrimer, composed of epoxy networks, could rearrange its topology and show viscosity variation through transesterification mechanism. After that, increasing attention has been attached to transesterification for exploiting more polymeric materials with ideal characters [67]. Nevertheless, emissive dynamic polymers based on transesterification reactions have rarely been reported. Recently, a blue emissive self-healing polymer composed of polyglycerol and itaconic anhydride was synthesized by Shan et al. and the fluorescence intensity of corresponding polymers could be selectively quenched by Fe^3+^ [68]. However, in this system there are no regular relationships between the transesterification and the fluorescence behavior of the polymer. As mentioned above, the transesterification reaction could significantly affect the mechanical performance of the polymers. Therefore, it is meaningful to realize the visualization of the transesterification in the polymer system. The Cui group reported a dynamic polymer based on transesterification which showed fluorescent color exchange as the transesterification reaction took place [69] (Figure 7). In this work, perylene diimides were linked by a spacer of dynamic ester to fabricate the dynamic polymer. Owing to the π-extended structure of perylene diimides, they stacked in a folding manner in the polymer system which exhibited orange emission. In the presence of benzenesulfonic acid, the transesterification reaction was effectively carried out under elevated temperature. During the transesterification process, the perylene diimides transformed from folding excimer state to the unfolding mono state, resulting in the fluorescent color being changed from orange to bright yellow. Structure usually determines the properties of a system. This work ingeniously designed a polymer by employing perylene diimides as the constructive unit. Owing to the different stacking modes of perylene diimides showing up in the polymer, it can be used to monitor the transesterification, which should provide guidance to design more novel dynamic polymers for understanding the mechanism of the DCBs working in the polymeric materials.

## 6. Polymeric Emissive Materials Based on Diels–Alder Reaction

**Scheme 6:**
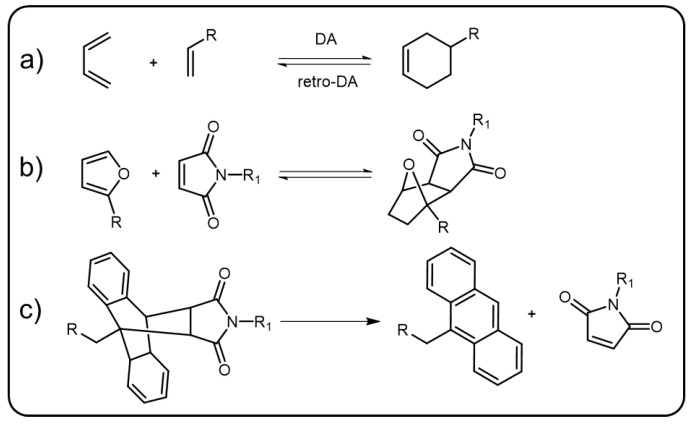
(**a**) The general formula of reversible Diels–Alder reaction. (**b**) Elevated temperature-induced reversible DA reaction between furan and maleimide derivatives. (**c**) Force-induced retro DA reaction between π-extended anthracene and maleimide derivatives.

The Diels–Alder (DA) reaction, named after Diels and Alder, was first discovered in 1928 [70]. It refers to a [4+2] cycloaddition reaction where an electron rich diene and an electron deficient dieniphile as the reactants form a cyclohexene product (Scheme 6a). The DA reactions are generally reversible in nature and the retro DA reactions usually require elevated temperature to take place. The cycloaddition between funans and maleimides (Scheme 6b) is one of the most representative DA reactions, in which the equilibrium moves forward at room temperature but backward at about 110 °C [71]. Besides funans and maleimides, the DA reaction can also take place between π-extended anthracene and maleimide derivatives to form five-membered ring products which are more stable under elevated temperature compared to the cycloaddition products of funans–maleimides [72]. To cleave the DA bonds of anthracene–maleimide derivatives, or in other words, to trigger the retro DA reaction in anthracene–maleimide derivatives, the temperature usually needs to be heated to 200 °C. External force, similar to elevated temperature, can also trigger the retro DA reaction of anthracene–maleimide derivatives in a polymer system (Scheme 6c). The DA bond, as a fundamental linkage, was used to construct polymer networks dating back to 1979 [73]. Since then, numerous polymers based on DA bonds have been reported. The reversibility of the DA reaction has made it the earliest chemical method to fabricate dynamic covalent polymers with a self-healing property [74]. By embedding fluorescence moiety into the DA-based polymer, stimuli-responsive emissive materials can be developed.

**Figure 8:**
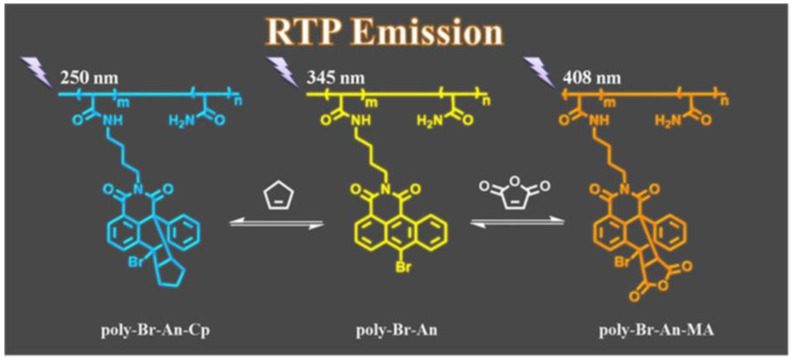
The structures of three organic room-temperature phosphorescence polymers in Ref. [81]. Reprinted with permission from Ref. [81]. Copyright 2020, Wiley-VCH.

Due to the easy formation under mild conditions and the reversible property at elevated temperature, DA bonds have been used to develop various kinds of thermal-stimuli-responsive luminescent polymers. In 2017, Mutlu et al. reported a thermally driven self-reporting polymer released system [75]. In this system, pyrene units were modified to parent polymers through DA reactions. There were some nitroxide radicals which could quench the luminescence of the pyrene units linked to the polymer chains. Thus, the emission of the polymer was silent under ambient condition. When treated with elevated temperature, the pyrene units were released from the polymer, resulting in the fluorescence enhancement of this system and the fluorescence intensity maxima was located at 397 nm. Additionally, the Martin group prepared several DA based π-conjugated polymers and studied their thermal-triggered optical properties in detail. First, they grafted oligo-(phenylene ethynylene)s (OPEs)-based π-conjugated donor and acceptor to two polymer chains via DA reaction, respectively [76]. Mixing these two polymers and heating up to 67 °C for several days, the mixed donor–acceptor grafting polymer was obtained. Owing to the fluorescence resonance energy transfer from the donor to the acceptor, the side chain grafting polymer showed efficient emission of the acceptor even if excited by absorption maximum of the donor. They further introduced the π-conjugated donor and acceptor into the polymer main chain [77]. In this work, bis-furan-modified OPEs served as the monomer to addition to a bis-maleimides-functionalized linker to form a copolymer through DA reaction. Similar to the side chain grafting polymer, the main chain polymer consisting of donor and acceptor could also display reversible exchange of the incorporated fluorophores under elevated temperature, and then showed efficient fluorescence resonance energy transfer. After that, the photophysical properties of such thermally reversible DA main chain polymers were carefully investigated further [78]. Very recently, increasing attention has been devoted to thermal reversible DA-reaction-based emissive polymers. For instance, Jiang et al. used a non-emissive dimaleimide-substituted tetraphenylethene (TPE-2MI) to link with three furfuryl-methacrylate-grafted copolymers through DA reactions [79]. TPE-2MI showed non-emissive behavior in solid state, which could be attributed to the photo-induced electron transfer (PET) between the tetraphenylethene and maleimide. By virtue of such a PET process, the synthetic polymer could show fluorescence switchable behavior depending on the temperature. At elevated temperature, the retro DA reaction took place and the fluorescence was quenched by the PET effect. On the contrary, the emission recovered at low temperature since the DA bond reformed. These polymers responded to the environment temperature in a fast and accurate manner, which could be applied to temporary information transmission. Utilizing the same strategy, the Jiang group embedded furan-containing polymer and TPE-2MI onto a PDMS substrate to form a bilayer system which showed tunable fluorescence and wrinkle pattern [80]. By virtue of such thermal sensitivity of the DA bond, DA-bond-regulated phosphorescent polymer has been also reported. The Ma group synthesized three organic room-temperature phosphorescent polymers (poly-Br-AN, poly-Br-AN-CP, and poly-Br-An-MA) which could be reversibly transformed via thermally reversible DA reaction [81] (Figure 8). The phosphorescent quantum yields of poly-Br-AN, poly-Br-AN-CP, and poly-Br-An-MA were 12.3%, 2.9%, and 8.4%, respectively, and the phosphorescent life times were 0.261, 0.219, and 0.256 ms, respectively. These polymers, showing different emission colors, could mutually transform under thermal conditions through DA reaction when different dienophiles were added into the system. The discrepancy emission colors of these polymers could be attributed to the different orbit energy and steric repulsion of the cycloaddition groups. This work tuned the phosphorescence emission by employing dynamic DA reaction into a polymer network, which could broaden the application of dynamic covalent chemistry in the photochemical field as well as provide new ideas for developing conspicuous emissive polymer materials with phosphorescence.

Additionally, combining thermal driven self-healing polymers based on DA bonds with fluorophores can also fabricate dynamic polymeric luminescent materials. Tatsi et al. reported a self-healing crosslinked polymer [82], which was obtained through linking a furan-modified acrylic copolymer and an aliphatic bismaleimide. Physically doping perylene-based fluorophore into the polymer, a luminescent solar concentrator system could be built. The optical efficiency of the luminescent solar concentrator decreased when the polymer film was damaged by external mechanical force. After thermal treatment, the optical efficiency could recover owing to the thermal healing of this DA-based polymer. Very recently, the Xu group constructed a thermoreversible polymer composed of thermoplasitic polyurethane and poly(ε-caprolactone) [83]. In this work, furan and maleic anhydride group containing cellulose nanocrystallines were integrated into the polymer to provide reversible DA-reactive sites while red fluorescent agent and carboxylic multiwall carbon nanotubes were embedded into the polymer to act as the emissive unit and UV shielding element, respectively. Stretching the composite polymer film, microcracks would form immediately and the fluorescent agent would be exposed to the ambient condition, enabling the absence of the UV shielding effect. Thus, the polymer film displayed red emission under UV light. Thermal treating and then cooling to room temperature, the microcracks were healed, resulting in the emission fading. The fluorophore-doped polymer containing reversible DA bond should be ideal material in damage self-reporting.

**Figure 9:**
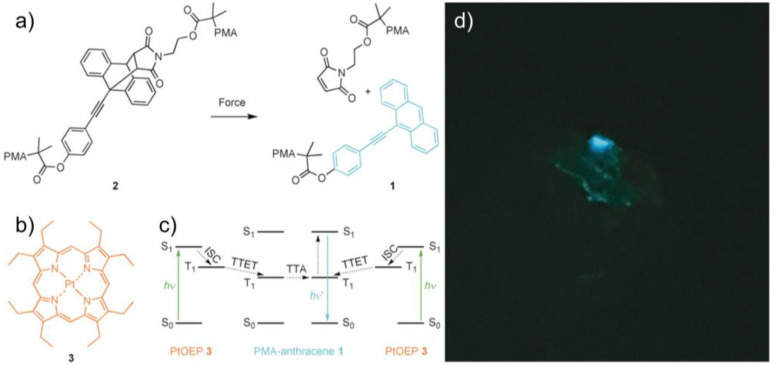
(**a**) Retro Diels–Alder reaction of PMA-substituted anthracene–maleimide to yield the PMA-substituted induced by mechanical force. (**b**) Triplet sensitizer octaethylporphyrin (PtOEP) used in Ref. [89]. (**c**) Jablonski diagram of the TTA-UC process in which 1 served as annihilator and emitter and 3 as the triplet photosensitizer. (**d**) The blue UC emission of the PHMA in solid state when swelled in a PtOEP solution. Reproduced with permission from Ref. [89]. Copyright 2019, Wiley-VCH.

Anthracene can be seen a diene to react with suitable dienophiles (maleimide derivatives), forming [4+2] DA cycloadducts [84]. During the formation process of the cycloadducts, the conformation of the anthracene transforms from the planar into twist state. When external force is applied to the adducts, the retro DA reaction will occur and facilitate the anthracene to return to the planar π-extended state which is emissive nature. Due to the mechanoluminescence, anthracene–maleimide, acting as the polymer linker sensitive to mechanical force, has been gradually studied [85,86,87]. In 2016, linear poly(methyl acrylate) (PMA) and crosslinked poly(hexyl methacrylate) containing anthracene–maleimide were developed by the Sijbesma group [88]. In this work, the π-extended anthracenes could be released from the anthracene–maleimide adducts via applying mechanical force to the polymer, both in the solution and solid state, and further showed blue emissive feature. Hence, the fluorescent π-extended anthracene releasing system could be used as a sensitive probe for mechanical force. They further introduced triplet–triplet annihilation photon upconversion (TTA-UC) into the mechanical sensing system (Figure 9) [89]. 9-phenylethynylanthracene, a known emitter in TTA-UC systems, was modified to PMA and then linked to a maleimide-group-terminated PMA through DA reaction. Adding the triplet sensitizer octaethylporphyrin to the as prepared poly(hexyl methacrylate), a mechanical force induced TTA-UC emission of the 9-phenylethynylanthracene could be detected and the upconversion quantum yield was 0.0823%. Very recently, the Li group adopted the same strategy to prepare a mechanical sensing polymer showing two distinct emissions [90]. These two emissions derived from the anthracene and amino-substituted maleimide, respectively. The maleimide fluorophores exhibited fluorescence quenching in protic or polar solvent, enabling dual emission behavior from this single polymer system in different solvents. All these DA-bond-linked polymers demonstrated above showed mechanical trigging emission behavior which might be applied to detect the damage of functional polymers. It is interesting that the dynamic break and reform of the DA bond can adjust the conjugation, even the electron distribution of the polymeric material, which is superior to other dynamic bonds, such as imine and boronic ester bonds, for regulating the optical properties of polymers.

## 7. Polymeric Emissive Materials Based on Dynamic C-C Bond

**Scheme 7:**
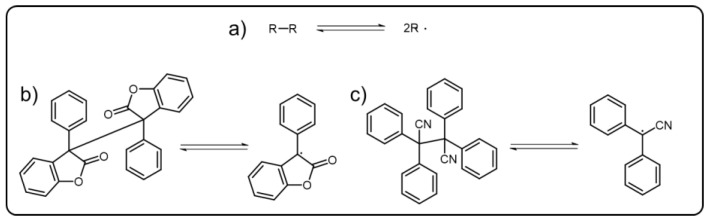
(**a**) Schematic representation of reversible dynamic C-C bond. The typical central radicals derived from (**b**) DABBF and (**c**) TASN.

**Figure 10:**
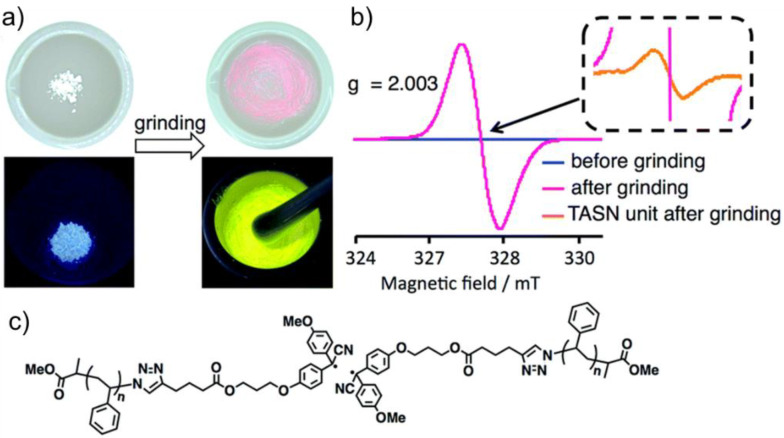
(**a**) Photographs of PS-TASN-PS before and after grinding, and the ground sample under UV irradiation. (**b**) EPR spectra of PS-TASN-PS before and after grinding. (**c**) Chemical structure of radical species derived from PS-TASN-PS. Reproduced with permission from Ref. [95]. Copyright 2022, Royal Society of Chemistry.

C-C bonds are generally regarded as irreversible and stable. However, the triphenylmethyl dimer itself was found that could undergo break and reform reaction via reversible recombination and homolytic cleavage of a C-C bond to form triphenylmethyl radicals [91], which, facilitating the dynamic C-C bond, gradually come into our sight. Actually, the dynamic C-C bonds refer to reversible δ_(C-C)_ bonds (Scheme 7a) which can undergo association and dissociation reaction under mild condition without catalysts. For achieving the dynamic C-C bond, the stabilization of the radical state through promoting spin-delocalization is significant. Introducing bulky substituents to protect the spin center and extending the π-systems to participate in the spin delocalization have proved to be effective methods [92,93]. As most DCBs, dynamic C-C bonds have been used as the reversible linkers of the polymers to build versatile dynamic covalent polymers. Diarylbibenzofuranone (DABBF), a typical molecular containing central dynamic C-C bond (Scheme 7b), has been widely incorporated into the polymer structures [94]. When the C-C bonds homogeneously cleave under stress, the blue radical derived from diarylbibenzofuranone can be obtained. There are some similar molecules that can also show color change behavior as their dynamic C-C bonds are cut off, such as tetraarylsuccinonitrile (TASN) (Scheme 7c), diarylbibenzothiophenonyl, difluorenylsuccinonitrile and diarylbiindolinone, etc. [95,96,97,98]. TASN, the unique one among these mechanochromic molecules, could present a luminescent behavior when transformed into corresponding radicals, which was first reported by the Otsuka group (Figure 10) [95]. The white powder of the TASN became pink and showed yellow luminescence under external mechanical force, and the absolute photoluminescence quantum yield was measured to be 39%. The pink color gradually disappeared within a few hours and reappeared under grinding, which could be attributed to the recombination and homolytic cleavage of the central C-C bond in TASN. By taking advantage of this unique feature, they embedded the TASN moieties into polystyrene chain which is glassy nature. When external mechanical force was applied to the copolymer (PS-TASN-PS), the copolymer changed from white to passion pink and exhibited a strong yellow emission with the emission peak at 560 nm. Owing to the fact that the rigid matrix could restrict the motion of the polymer chains and further suppressed the recombination of the radicals, the yellow emission could remain for several months. Utilizing the mechanoluminescence of TASN derivatives, freezing-induced luminescence of TASN-containing polymers was also constructed by the Otsuka group [99,100]. In this regard, TASN-containing polymers could be applied to a highly sensitive probe for mechanical stress owing to its optimal mechanoluminescence property. Similar to the anthracene–maleimide-based DA bond, the introduction of a dynamic C-C bond endows the system with mechanoluminescence property. The reform process of the dynamic C-C bond could occur under milder conditions and faster than a DA bond, which makes it an alternative for practical application.

## 8. Conclusions and Perspective

Compared with the robust covalent bonds, DCBs are not only stable under ambient conditions, but also show reversible associative and dissociative behavior under external stimuli. Therefore, the DCBs can be easily introduced into polymer materials, endowing the dynamic polymer materials with versatile properties beyond the common ones. In this review, we summarized the reaction mechanisms and representative features of various DCBs-based polymers, as well as showed their emission properties. Physical doping and chemical modification of luminophore into the dynamic polymers are both effective strategies to build dynamic covalent polymers with emissive property. These dynamic emissive polymers are stable under ambient condition, and they show good stimuli responsiveness, such as pH (imine bond, acylhydrazone bond, boronic ester bond), temperature (Diels–Alder reaction, transesterification), and mechanical force (anthracene–maleimide-based Diels–Alder reaction and dynamic C-C bond). Moreover, some DCBs-based dynamic emissive polymers can also display self-healing and reconfigurable behaviors. Combining the emission and self-healing features, the application of such polymeric materials might be greatly extended. Interestingly, there are efficient bond exchange (imine bond, acylhydrazone, transesterification) processes which happen in some emissive dynamic polymers resulting in tunable emissive behavior of these polymers. DCBs (imine bond, acylhydrazone bond, boronic ester bond) which are reversible and possess effective error correction capability can also serve as linkers to develop more highly emissive COFs. These DCBs-based emissive polymeric materials may help people to have a deeper understanding of dynamic covalent polymers with emission.

As time passes, increasing attention has been devoted to emissive materials owing to their potential applications for bioimaging, optical displaying, etc. Besides emissive polymers, non-polymeric emissive materials such as inorganic emissive glasses (doped with ions), as well as small organic emissive materials, have also been widely studied. Ions (iron, cobalt, rare-earths, etc.) are doped into the glasses, which can endow the glasses with multi-color emissive properties and control the carriers transport of the semiconductors. However, this method is hard to precise control of nanostructured architectures, which may hinder the further design of these materials [101]. For the small organic molecular emissive materials in the sensing field, the signals of such materials can be easily detected but these signals are relatively weak. To accommodate this, it is a feasible method to amplify the sensing signal by polymerization of these sensing monomers [102]. Compared to these two materials, the structures of DCBs-based PEMs can be easily regulated and the emissive performances of the PEMs are better. In addition, crystal-based emissive materials whose inner structures can be easily obtained enable the better understanding of the structure effect. Nevertheless, compared to polymeric emissive materials, crystal-based emissive materials are hard to prepare on a large scale and hard to process, which greatly impedes their further applications [103]. We believe that the DCBs-based PEMs which possess optimal and controllable emissive properties are ideal candidates to be applied in the field of emissive materials. Despite many efforts being devoted to fabricate PEMs based on DCBs, further research is still necessary. Polymeric materials are generally crosslinking and solid nature, which may limit traditional emissive fluorophores being used in such conditions. The AIEgens are suitable candidates which can display distinctive emission behavior in aggregate for achieving the bright emission of polymeric materials in aggregated and solid states. Tetraphenylethylene, a typical AIE molecule, is one of the most important units that has been used to fabricate such polymeric materials. Taking the fluorescence diversity into consideration, AIEgens with multicolor emission can also be integrated into the polymeric materials to regulate the emission wavelength of the PEMs. Similarly, quantum dots and nanoclusters can also be ideal fluorophores for developing emission-color-tunable PEM hybrids owing to their emission behavior, which can be easily adjusted by changing their constitutions or dimensions. Besides the additive fluorophores, the break and reform of DCBs may change the conjugated structure or charge distribution of polymeric materials and further affect the optical performance of such polymers, which can be considered as a rational strategy to tune the emissive properties of PEMs.

In addition, the extreme physical/chemical stimuli such as thermal, pH, and force, etc., used to trigger the association and dissociation of the DCBs, may affect the luminescence of PEMs to some extent and hinder the application of these materials. Hence, introducing more commonly used DCBs which are reversible under mild stimulus into the luminescent polymers can extend the categories of PEMs. For instance, disulfide bonds, pyrazole-urea bonds, and oximes bonds, etc., can be considered as potential candidates for constructing such intriguing PEMs owing to their good dynamic property to tune the inner structure of polymeric materials [104,105,106]. Because different DCBs are sensitive to different external stimuli, by integrating two or more DCBs into one polymer system, the polymer system may show different emissive behaviors under orthogonal stimuli [107]. Therefore, it is a rational strategy to develop multi-responsive PEMs by integrating orthogonal DCBs into the emissive polymer systems.

Owing to the excellent performance of the DCBs-based PEMs, versatile applications can be proposed. On the one hand, some DCBs-based PEMs are stretchable and highly emissive, and such material can be used to fabricate wearable and flexible photoelectric devices. Additionally, the stimuli-responsive features make them convenient for developing smart materials. For example, pH sensitive materials could be suitable to be used in the bioimaging field, and thermo-responsive materials might be devoted to information storage. In addition, mechanoresponsive materials should play a key role in the detection of damage to functional polymers in a visible manner. On the other hand, the easy processing and good adaptivity of DCBs-based PEMs can be used for recyclable painting. Recently, polymeric circularly polarized luminescence (CPL) materials, as rising photoelectric materials, have been widely studied [108]. Introducing DCBs into these polymers can enrich the database of such materials and help us uncover the corresponding relationship between the polymer structure and CPL. In this regard, increasing attention needs to be attached to such outstanding materials and we believe that further promoting the performance of the DCBs-based PEMs will provide a potential platform for practical applications.

## Data Availability

Not applicable.
